# Building from Patient Experiences to Deliver Patient-Focused Healthcare Systems in Collaboration with Patients: A Call to Action

**DOI:** 10.1007/s43441-022-00432-x

**Published:** 2022-07-19

**Authors:** Karlin Schroeder, Neil Bertelsen, Jessica Scott, Katherine Deane, Laura Dormer, Devika Nair, Jim Elliott, Sarah Krug, Ify Sargeant, Hayley Chapman, Nicholas Brooke

**Affiliations:** 1 Parkinson’s Foundation, New York, NY USA; 2 Health Technology International (HTAi) Patient and Citizen Involvement Interest Group, Berlin, Germany; 3 Takeda, Cambridge, MA USA; 4 School of Health Sciences, University of East Anglia, Norwich, UK; 5 Future Science Group, London, UK; 6 Vanderbilt University Medical Center, Nashville, TN USA; 7 Health Research Authority, London, UK; 8 CANCER101 Inc, New York, NY USA; 9 Health Collaboratory, New York, NY USA; 10 Twist Medical, Burlingame, CA USA; 11 Patient Focused Medicines Development (PFMD), The Synergist, Brussels, Belgium; 12 Patient Focused Medicines Development (PFMD) Patient Engagement and Patient Experience Data Project Team, Brussels, Belgium; 13 Present Address: Legacy Health Strategies, Del Mar, CA USA

**Keywords:** Patient experience data, Patient-focused healthcare, Collective value patient experience

## Abstract

Patients’ experiences of their diagnosis, condition, and treatment (including the impact on their lives), and their experiences surrounding expectations of care, are becoming increasingly important in shaping healthcare systems that meet the evolving needs and priorities of different patient communities over time; this is an ongoing goal of all healthcare stakeholders. Current approaches that capture patient experiences with data are fragmented, resulting in duplication of effort, numerous requests for information, and increased patient burden. Application of patient experience data to inform healthcare decisions is still emerging and there remains an opportunity to align diverse stakeholders on the value of these data to strengthen healthcare systems. Given the collective value of understanding patient experiences across multiple stakeholder groups, we propose a more aligned approach to the collection of patient experience data. This approach is built on the principle that the patients’ experiences are the starting point, and not just something to be considered at the end of the process. It must also be based on meaningful patient engagement, where patients are collaborators and decision makers at each step, thereby ensuring their needs and priorities are accurately reflected. The resulting data and evidence should be made available for all stakeholders, to inform their decision making and healthcare strategies in ways that meet patient priorities. We call for multi-stakeholder collaboration that will deliver healthcare systems and interventions that are better centered around and tailored to patient experiences, and that will help address patients’ unmet needs.

## Introduction

Healthcare systems aim to improve the health of populations, and to transform the lives of patients. Research increasingly demonstrates that patient-centered (or patient-engaged) care achieves the best outcomes, and there is a global movement toward this model of healthcare [1–4]. Crucially, this type of care concentrates not only on important clinical outcomes but also on improvements in wider outcomes that patients say matter to them, and which may not have previously been given the prominence they deserve. Outcomes of interest to patients include improvements in health-related quality of life or functional outcomes, patient, caregiver, and family experiences, healthcare resource utilization, and care provider satisfaction. Concepts of engagement, communication, quality, and safety are fundamental matters for healthcare that drive decisions in healthcare systems around the world, and they all converge in the experience dialogue [5]. To achieve desired goals of treatment, all segments of healthcare and all stakeholder groups need to understand and learn from patient perspectives and experiences, and to use these insights to develop strategy and inform decision making. The research that leads to, and results from, these insights must be designed and implemented in partnership with patients. Patient engagement can optimize research by ensuring it reflects the needs and priorities of patients in a way that is least burdensome to them [6]. This article focuses on the collective value of patient involvement and proposes an approach to healthcare systems that starts with the patient experience and is co-created with patients.

## Patient Experience and Patient Experience Data

Meaningful patient perspectives can be collected and contextualized into data for use by stakeholders across healthcare systems. These data cover the entire patient (and caregiver and/or family) experience of being diagnosed with, living with, or being treated for a disease or condition; they also incorporate the “customer” experience of using a healthcare system. Patient experience data (commonly referred to as PED but PXD will be used in this article) have been defined as, “data that are collected by any persons and are intended to provide information about patients’ experiences with a disease or condition. PXD can be interpreted as information that captures patients’ experiences, perspectives, needs, and priorities related to (*but not limited to*): (1) the symptoms of their condition and its natural history; (2) the impact of the condition on their functioning and quality of life; (3) their experience with treatments; (4) input on which outcomes are important to them; (5) patient preferences for outcomes and treatments; and (6) the relative importance of any issue as defined by patients” [7]. This definition is intentionally broad and includes patient survey data, patient-reported experience measures (PREMs), patient-reported outcome measures (PROMs), patient focus groups or meeting reports, patient registry data, patient preference data, clinical outcome assessment (COA) data collected as part of clinical trials, and natural history study data. PXD therefore aims to provide information that fully captures patients’ experiences, perspectives, needs, and priorities, which can then be used by stakeholders to co-create research with patient communities and inform healthcare decisions. Essentially, it is the holistic patient experience of both non-clinical and clinical outcomes as well as the process of partnering with patients that are the cornerstones of this endeavor; these are more important than the data collected or the users of the data.

It should be noted that simply gathering or using PXD is not necessarily synonymous with being patient-engaged. For example, the term “PROMs” implies that the questionnaires are capturing what is most important to patients and that they have even been developed with their input, when this is seldom the case. There is now a drive to design PROMs with patients involved throughout the process; however, there are many in routine use that are well validated but that do not capture the exact nature of outcomes that patients have identified as priorities for them. These PROMs mostly encapsulate what the clinicians and researchers thought were important to patients at the time of developing the questions [8, 9]. The Consensus-based Standards for the Selection of Health Measurement Instruments criteria is an initiative that highlights the need for, and importance of, PROMs that are based on patient input [10, 11]. In addition, programs such as the COMET initiative have emphasized the need to select appropriate outcomes for clinical trials that are relevant to all stakeholders, including patients [12].

Furthermore, patients’ experiences and expectations change as they move through different stages of their disease, possibly dealing with additional comorbidities and changing life circumstances. Standards of care evolve also. While this article goes on to argue that it is important to avoid duplication of efforts in gaining PXD, this does not mean that collection of data is only performed once; rather, it should be an ongoing process and revisited as needed, to reflect the evolving patient experience.

Patients would work with data scientists, (biostatisticians, bioinformatics, quality analysts, clinimetricians) and, in the case of medical technology and device industry, development teams to ensure data are both usable and patient-centered/patient-driven. Scientists ensure data are scientifically and ethically sound by applying their professional expertise in statistical design and methodology; patients ensure that data that are most important to the community is prioritized for collection and is ethically collected in the least burdensome way possible. This means patients must be included early in study design, from the study concept stage and at every step of the process as part of the study team.

Patients must also be included as part of the study team in creating new clinical outcomes assessments, developing and deploying surveys, selecting clinical outcomes assessments, developing new devices including feasibility and usability studies, monitoring the data, interpreting the data from a community perspective, and ensuring the results of the study are disseminated in the community in lay summaries. This may mean training patients in understanding issues around data in research and care, and training researchers and clinicians in patient engagement. It is likely that data will be more usable through this collaborative approach because the study was well designed from a community perspective. Ideally, if the study is less burdensome to the community and research teams, and more focused on community priorities, there would be less missing or poor-quality data.

Furthermore, not every condition has a patient organization and some (nascent) organizations may be very small and minimally funded. As such, collaboration is key as capabilities may differ across conditions. There may also be many patients wanting to contribute to advance research and development who are not "activated" patients and part of a patient organization. A systematic approach to involving patients, increasing co-creation, sharing lessons learned and emerging best practices from each stakeholder perspective and the value derived from upstream work to downstream impact is essential to support an ongoing, dynamic, and adaptable process of PXD collection and use.

## The Collective Value of PXD Across Diverse Health Stakeholder Groups

There is a common need for PXD that informs evidence-based decision making among diverse groups of stakeholders; however, information is collected individually rather than collectively, and not usually in partnership with patients. An approach to develop and collect PXD in a collaborative manner would result in a collective value of that data for all stakeholders in the healthcare system, and this is recognized globally. For example, in the UK, the National Institute for Health and Care Excellence has acknowledged the significance of patient preference studies in informing health technology assessments (HTAs) [13]. There is growing evidence of the impact that patient involvement can have on HTA deliberations, such as providing context, new information, and reassurance [14, 15]. The importance of working alongside patients to improve the methods by which their input is gathered and considered for HTA discussions has also been highlighted [16]. The US Food and Drug Administration (FDA) has described the patient perspective as “critical” for regulatory decision making and has established Patient-Focused Drug Development public meetings to hear directly from patients and their families, caregivers, and patient advocates [17]. The Canadian Agency for Drugs and Technologies in Health (CADTH) has had a long-standing commitment to incorporate patient and public perspectives in HTA; it provides regular examples of how patient input had a tangible impact on CADTH reviews, providing much-needed feedback that documents the value of these efforts [18]. These insights are used to inform healthcare system decision making, including funding decisions and prioritization.

The FDA has also noted the benefits of engaging with patients to design and conduct research [19]. The richness of this type of qualitative data can be valuable if collected robustly and could be used by different stakeholders, thereby preventing duplication of effort. Another example is that of the James Lind Alliance and its use of Priority Setting Partnerships that bring together patients, caregivers, and clinicians to agree key priorities for future research [20].

The collective value of PXD is reflected by the increasing requests for and use of these data across different healthcare stakeholder groups, such as patient advocacy organizations, regulators, HTA organizations, and pharmaceutical companies [19, 21, 22]. Although the PXD can be used by different stakeholders and for a variety of purposes, the methods, range, and type of questions that need to be asked to gather the data are overwhelmingly similar. PXD questions focus on core themes that look at patient experiences with the burden and impact of their condition and their treatment (e.g., emotional, psychosocial, employment, family, and other effects), as well as their expectations for better health outcomes (Table 1). Broader, more holistic PXD is also gathered by different organizations. For example, the UK conducts an annual National Cancer Patient Experience Survey covering the entire patient journey and the patient experience of cancer care. It asks how patients felt they were treated and listened to, whether they had their diagnosis or care explained in an understandable way, and what the care environment and care staff attitudes were like [23]. In addition, tools such as the Accountable Health Communities Screening Tool used in the United States, capture social determinants of health, based on the understanding that lifestyle issues (such as housing instability, transportation/access issues, and food insecurity) also impact health and health outcomes [24].

**Table 1:** Different stakeholders are asking for the same kind of PXD

Common themes	PXD requests from different stakeholder groups^a^ [19, 21, 22, 25]
Burden and impact of disease/condition	What are the signs and symptoms patients experience and how do these affect their day-to-day functioning and quality of life? How does this condition affect the day-to-day lives of people living with it in terms of challenging symptoms and activities that patients find difficult or unable to do? What are the aspects of the condition that are most important for patients to control? What disease effects matter most to patients that might be addressed by a medical therapy? What is the course of their disease over time, including the effect of the disease on patients’ day-to-day function and quality of life over time, and the changes that patients experience in their symptoms over time?
Burden and impact of treatment/trials	What are patients’ experiences with the treatments for their disease? How well do current medicines help patients manage this condition? What are the benefits/risks of current treatments, both in the short and long term? What is the burden of treatment (including the effect of treatment on activities of daily living and functioning) and/or the burden of participating in clinical studies? What treatment burdens matter most to patients that might be addressed by a medical therapy? What would be the best way to measure these disease or treatment burdens/effects in a clinical trial? What would be the most appropriate endpoints to use in clinical trials (and robust enough to inform regulatory decision making)? What is a clinically meaningful change in an endpoint from a patient perspective? How to define meaningful change in a patient over time?
Expectations for new (and current) treatments	What are the patient expectations of benefits? What are the potential QoL improvements and/or health outcomes? What aspects of patient needs or expectations could the new treatment potentially address? What are the potential impacts on caregivers and family such as potential reduced dependency and emotional and psychosocial impacts? What are acceptable trade-offs of benefits and risks (i.e., patient preference)? What methods and approaches could be used to identify which treatment benefits would be most desirable to obtain and which risks would be most important to avoid, or to explore what patients might consider to be acceptable trade-offs of increased expected harm(s) for a specified increase in expected benefit with a new medicinal product? What are patient attitudes towards uncertainty? What are patient views on unmet medical needs and available treatment options?
Emotional, psychosocial, employment, family, and other effects [25]	How are patients feeling in terms of pain or discomfort, feeling low or worried, limited in what they can do, requiring help from others? How are patients feeling in general? How well do patients look after themselves? Have patients learned to live with what has happened? Thinking about way of life, such as education, feeling valued for what they do, being happy where they live, having enough money to cope—how do patients feel? Thinking about family and friends, having people to talk to, having someone to confide in, having people who will help—how do patients feel? How often do patients feel left out, alone, or lonely? How safe do patients personally feel at home and outside of home?

^a^Examples from regulators, health technology assessment organizations, and pharmaceutical companies, as well as PROMs or PREMs

*PREMs* Patient-reported experience measures, *PROMs* Patient-reported outcome measures, *PXD* Patient experience data, *QoL* Quality of life

There have been calls for clarity and direction for the collection and use of PXD; this has led to the development of guidance for PXD, further demonstrating its growing importance in healthcare decision making. For example, Ensuring Value in Research (an international body comprised of organizations that fund health-related research or establish funding policy) has produced guidance that focuses on increasing the value of research, which includes patients when referring to the “meaningful involvement of those who will use and be affected” by the research [26]. Another venture is the European-wide PREFER program, a public private partnership research project under the Innovative Medicines Initiative, which aims to provide recommendations to support guidelines for industry, regulatory bodies, and HTA organizations on how and when to assess and include patient preferences in medicines development [27]. The Scottish Medicines Consortium has issued guidance for patient group partners who want to submit evidence to them; the guidance can be incorporated into the overall discussions about the clinical benefits and cost-effectiveness of new medicines [21]. Patients and patient advocacy organizations have also called for PXD collection to be streamlined and harmonized. They recognize there is a need to lessen the burden on the patient community of repeating information to multiple stakeholders, and to reduce the overuse of resources dedicated to the repeated collection of the same information for pre-competitive purposes.

## Starting PXD Collection Based on Stakeholder Perspective is not Appropriate

Despite the goal of achieving more patient-engaged healthcare and the similarities in the types of data being requested by different stakeholders, in general, current approaches to PXD do not have the patient at their center and lack stakeholder alignment. Instead, strategies are developed from each stakeholder’s perspective in silos and then “backfilled” with PXD by each stakeholder. Although there are efforts to develop a more harmonized approach, these are generally aimed at achieving alignment within one stakeholder group, for example from a regulatory point of view [22], rather than alignment that traverses several groups. In these cases, patient partners work with a stakeholder to put patient community needs and priorities at the forefront, but this partnership needs to be replicated for multiple stakeholders, and this puts intense resource and capacity expectations on the patient community.

There are several unwarranted consequences of a stakeholder-centric and fragmented approach to PXD. First, if healthcare systems are to be patient-focused and the aim of health interventions is to transform their lives, it is essential to identify from patients themselves what their expectations are. Capturing the human experience (including non-clinical perspectives) and using it to identify how the healthcare system can improve the individual and shared experiences of patients is key to patient-engaged healthcare. Secondly, each stakeholder group will have their own perspective; for example, regulators see challenges and solutions through a different lens to HTA organizations. As a consequence, stakeholders may inadvertently bring their own assumptions to the development of PXD (deprioritizing the patient perspective as less important) and overlook concepts that matter to patients. Thirdly, siloed approaches and a lack of collaboration mean that different stakeholders are asking for the same kind of PXD, thus causing duplication of effort and repetitive (and potentially onerous) requests to patients. Calls to reduce waste in research are numerous [28–30] and applicable to the current disjointed approach to PXD. Lastly, siloed and stakeholder-centric approaches that start from the perspective of each different stakeholder group lead to the generation of separate pieces of PXD (adding to inefficient use of healthcare system resources), which are biased by stakeholder views and lead to misaligned understanding of the true patient experience. This could subsequently impact healthcare decisions and systems that may be based on inaccurate PXD. For these reasons, all decisions in stakeholder groups should be made with the full understanding and consideration of the patient experience.

## Evolving from Stakeholder-Centric to Holistic and Patient-Engaged PXD

**Fig. 1:**
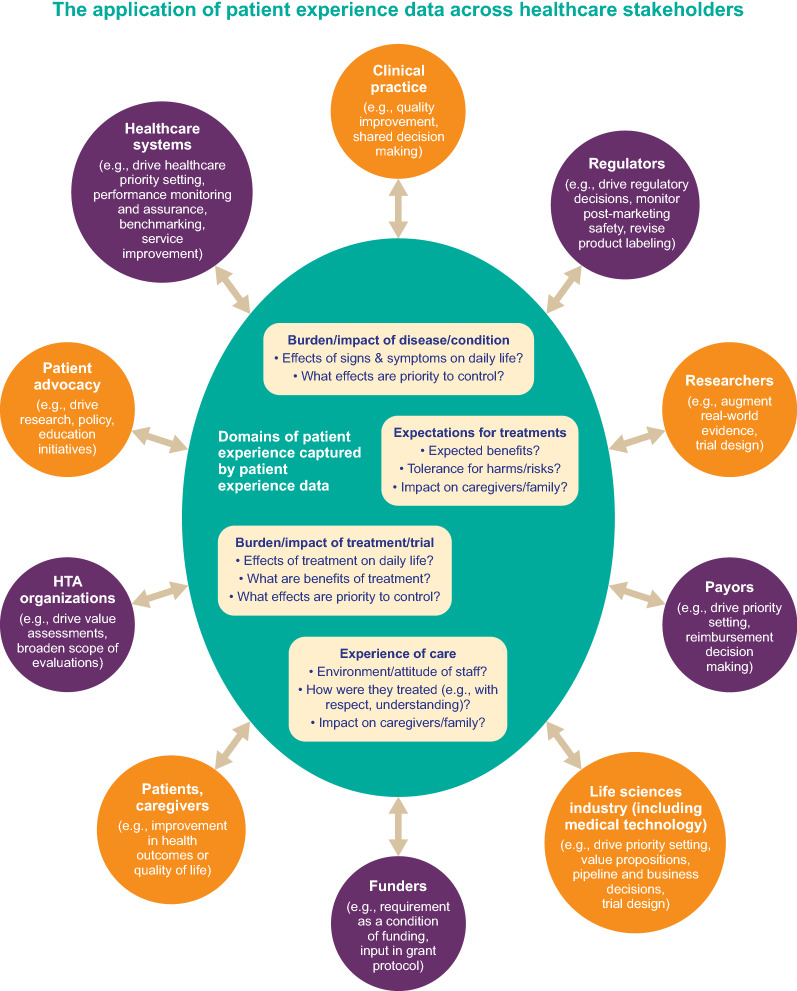
The collective value of PXD across diverse healthcare stakeholder groups provides opportunities for integration and a patient-engaged approach [19, 21, 22, 37–42]. *PXD* patient experience data

Different stakeholders have a shared need for the same information around the patient experience (Fig. 1). Common themes include how the patient was treated (e.g., with kindness, compassion, empathy, dignity, or respect), physical comfort (e.g., adequate and timely pain control), emotional support (e.g., being able to discuss anxieties and fears), communication (e.g., listening, explaining clearly, education, information), involvement of family and friends (e.g., opportunities for them to talk with the healthcare team), promptness (e.g., delays, cancellations, responsiveness, waiting times), organization (e.g., management of the service, co-ordination of care, continuity and transition, efficiency, reliability), and environment (e.g., safety, cleanliness, quietness, facilities) [31–36]. This collective value of PXD across diverse healthcare stakeholder groups provides opportunities for integration. In practical terms, where there is overlap or repetition in the PXD requested, stakeholders should work together, including with patients, to ensure that information that is of interest across stakeholders is only collected once (or as few times as possible) and is shared, to meet all stakeholders’ needs.

Although this may not always be possible in all situations, every effort should be made to identify synergies and minimize duplication. Where there is overlap in PXD, one way in which gaining consensus across disease could be approached could be through professional societies working with patients to set data standards that are acceptable to all stakeholders. In this case, it would be important to ensure it is a truly international collaboration with representative stakeholders driving the development of standards and methodologies.

## Mutual Benefits of a More Collaborative Approach to the Collection and Use of PXD

An aligned approach for the type of PXD and methods of collection would provide several opportunities for quality improvement; for example, by identifying and replicating best practice, or for benchmarking to help reduce unwarranted variation in the quality-of-service delivery. Lee et al. have proposed a model for designing a healthcare service based on the patient experience [43].

These shared needs for the same PXD (currently gathered in a repetitive and duplicative manner) highlight the requirement for a more collaborative and streamlined approach. Rather than working in vertical, unlinked silos, PXD should be thought of as “user agnostic”—having value and relevance for diverse healthcare stakeholders. Disparate but complementary PXD efforts should be brought together and PXD placed firmly at the center of healthcare systems so that insights from patient experiences drive healthcare strategy and improvements (rather than developing strategy that is not driven by patient experience insights, as is currently the case) (Fig. 1). A benefit of collaborative stakeholder working is that the whole will be greater than the sum of its parts, which means that what is achieved will be more accurate, of better quality and use fewer resources.

Leveraging PXD to inform healthcare decision making will benefit the whole healthcare system and help to address current misalignment between what patients need and what they receive. Developing a collaborative approach to defining and collecting PXD would deliver healthcare systems that are focused on improving outcomes that are most meaningful to patients and not only those valued by other stakeholders. For example, it was patient preference data that led to the development of a subcutaneous formulation of the traditionally intravenous drug rituxan [44]. Rituxan Hycela® was approved by the FDA in 2017 and may be the first instance of a “Patient Experience” section being included in a drug’s label [45]. PXD was also integrated into a new drug application (NDA) for a novel antidepressant; its inclusion contributed to the FDA’s decision to approve the application [46]. In June 2021, the European Medicines Agency (EMA) proposed guidelines for incorporating patient experience to better inform regulatory decision making [47], meaning that, like the FDA, it recognizes the importance of developing a PXD strategy early in medicines and devices development. Although around 70% of NDAs include PXD [37], not all PXD are created equal; many tools that capture patient experience have not been developed in partnership with patients [48] and the validity and reliability of different tools vary [49]. Tools or metrics to evaluate the impact of health-related conditions on patients should also consider immediate and longer-term impact. For example, the measure of absenteeism in the workplace does not fully capture, and may therefore underestimate, the impact of health on work [50]. Absenteeism also fails to capture negative career consequences such as having to work part time, enforced role change, or lost job opportunities. For PXD to be meaningful, actionable, supportive, and informative, it must be co-created with patients.

It would also be valuable to collaborate with the patient community on the data collection, or for the patient community to collect the data. While patient communities often use validated tools for data collection, some may need capacity building or training to achieve robust data collection capability. Similar to calls for data sharing amongst research teams, calls for data sharing amongst patient groups and patient advocacy organizations should be made. To reach diverse and underserved communities, a collaborative approach that uses multiple data collection methods that best meet community needs would be optimal. One example of patient community data collection is the National Kidney Foundation Patient Network registry in the United States combines data from electronic health records and patient-centered data [51]. The registry’s online platform has been designed to allow people with kidney disease to enter their own health information, which ensures that insights and data captured are truly reflective of the patients’ experiences.

It is important to acknowledge that there are shared challenges to the multi-stakeholder collaborative collection and use of PXD, including those involving culture, process, and lack of infrastructure and tools for PXD [37, 52–55]. However, by understanding the mutual benefit of PXD across diverse stakeholders, some of these challenges can be addressed. For example, identifying commonalities means that the risk and burden of generating PXD are shared across groups with the knowledge that other stakeholders will also value and use the PXD. In terms of process and operational aspects, cost and resource efficiencies can be gained by reducing duplication of effort and taking a more aligned approach to PXD. There is also the potential to improve the quality of PXD by having a more holistic and truly patient-engaged approach and precompetitive consortia would be ideally placed to address this, especially across multiple studies within a disease group or condition. Regardless of the existing barriers, it is neither desirable nor sustainable to continue developing medicines and medical devices that do not demonstrate that they meet the needs of patients. Just as effective use of PXD that is meaningful to patients can yield tangible benefits, failure to put patients at the center of healthcare decisions can have consequences. For example, the medical profession’s failure to acknowledge that women and men have different experiences with heart disease has led to women receiving suboptimal treatment and reporting poorer outcomes, including worse quality of life and more physical and psychological disability [56–59].

## Limitations of PXD

Despite its potential, PXD is not a magic bullet. For example, PXD covering work-related experiences, such as being able to work at all or working full time, do not reflect that many contributing factors that affect a person’s ability to work, e.g., societal attitudes, equality regulations, and laws, lie outside the area of influence of a drug or device. Health and illness are not dichotomous states, and some patients may never return to full health. The reality is that for most long-term conditions, the goal is improved management, reduction of symptoms, and prevention of future harms. These improvements may not equate to increased capacity or ability to work.

A further issue is data security, especially as PXD may be highly sensitive and personal. Despite anonymization or pseudonymization, there have been high-profile breaches of health data security [60] or use of algorithms to re-identify individuals [61]; incidents like these make people wary of who might have access to PXD (e.g., medical insurance, policing, or benefits agencies) and for what reasons. However, even with these concerns, many patients seem willing to disclose their data for the greater good as long as it can be shared safely [62–66]. Ongoing communication with patient communities about perspectives on data security is critical as the technology advances, to ensure data capture and storage methods are driven by patient preferences and rights. Additional consideration should be given to working with patient communities whose data have been misused or underdeveloped in the past, causing harm to communities that already experience health inequities due to systems that perpetuate disparities in care. The potential for security issues and undesirable uses of PXD will need to be carefully assessed, with safeguards put into place that are robust enough to withstand the political variations of privacy rights and other regulations across the world.

With advances in technology comes the ability to collect a multitude of data points, and this is another area for consideration. Just because vast amounts of data can be collected, it does not necessarily mean that it should be. It is important that sectors across healthcare have a clear and well-explained rationale for each data point that is being collected, what they will potentially do with the data point, and how it will potentially converge with other data points.

Finally, large data sets of PXD must accurately represent the patient population. Like most research, PXD has historically tended to lack demographic and disease-state/experience diversity, often reflecting only a subset of a population that is white and wealthy. Additionally, PXD that is self-reported and captured electronically risks ignoring communities that do not have internet access, digital, or technological devices, or technological literacy. Working with representative populations from a given patient community and including them as equal decision makers in the PXD development, as well as creating systems that allow those populations to easily contribute PXD, will be imperative to ensuring the data truly reflects whole patient communities and can be applied to improving healthcare equitably.

## What Does “Good” PXD Look Like?

There is a collective, shared value for PXD in the healthcare system and there are mutual benefits to be gained from collaboration. The next logical step is to identify an aligned set of needs and a roadmap to jointly and fairly collect high-quality PXD, without duplication of effort, for diverse stakeholder groups and multiple purposes. This should be driven with quality patient engagement [67] to ensure that the data collected are patient-engaged, meaningful, representative of diverse patient populations, fit-for-purpose, and, consequently, of value to multiple stakeholders. Sharing a PXD repository that is integrated and accessible across all stakeholders removes duplication and begins to build the capability of developing PXD in a synergistic and holistic way. There have been suggestions for how a repository of PXD could be captured using open sources of information but there are significant technical and logistical challenges that would need to be addressed [68–70].

**Fig. 2:**
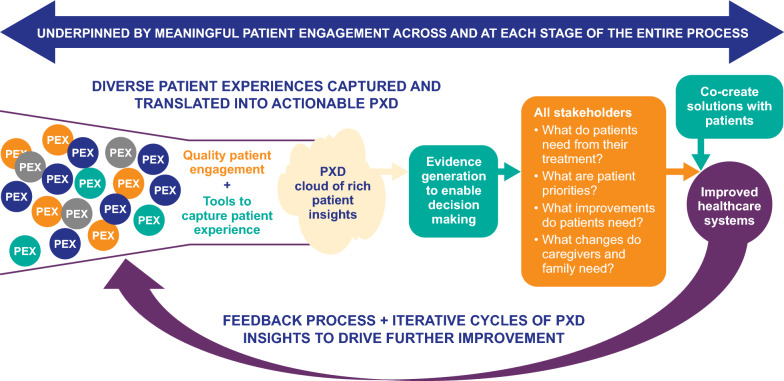
Cohesive approach to integrated PXD. *PEX* patient experience, *PXD* patient experience data

By taking a patient-engaged approach to PXD, we can assess the usefulness of existing tools (e.g., PREMs, PROMs, COAs), and identify gaps that are not currently measured, thereby prioritizing the development of new tools that can more accurately capture the breadth of patient experiences. It would also be beneficial to streamline and standardize the number and different types of tools that are used by identifying common underlying constructs [71, 72]. These tools should be carefully assessed for validity and reliability and also be regularly assessed and updated to meet current trends and needs within a patient population. [73–75]. The approach should also incorporate feedback mechanisms to ensure that how the PXD is used, and the impact of that use, is communicated to patients and that patient feedback is used to further improve how PXD is collected, stored, and employed. In addition, as healthcare improves due to better understanding of patient needs, expectations and practices will change and patient experiences will evolve. Patient feedback will be crucial to understanding and adapting to this evolving landscape (Fig. 2).

## Call to Action: Multi-stakeholder Collaboration and Partnership with Patients to Deliver High-Quality PXD

The range of stakeholders involved in healthcare interventions and systems means that multi-stakeholder collaboration is essential for the collection of PXD (in partnership with patients), which should be done as few times as possible and for multiple purposes. We call for all stakeholders to work together to co-develop a methodological roadmap for, and implementation of, an integrated approach to PXD. This framework would empower the whole system to address the unmet needs of patients more efficiently, holistically and consistently. We also call for the co-production and establishment of a shared and accessible repository for PXD and for increased high-quality patient engagement in PXD approaches. Such engagement should follow established (and freely available) guidance for meaningful co-production and collaboration with patients, including adherence to well-defined quality criteria for patient engagement [67].

The current call to action is not from a lone voice. The EMAs Regulatory Science Strategy to 2025 called for global alignment on the methodology used to gather PXD and patient contribution to medicines development [76]. Others have highlighted an “urgent need” for global co-operation and collaboration of HTA and research organizations to fulfill the potential of PXD in HTAs [77]. There are also patient-led collaborative efforts to support the development and use of PXD to broader applications and for a repository approach to PXD. For example, the US National Health Council is leading an initiative for the development of patient-engaged core impact sets [78] and is creating a Patient Experience Mapping Toolbox [79]. In the UK, UseMyData promotes a more central collection of patient data (focused on hospital data and statistics) that can be used for varying purposes [80].

## Conclusions

In summary, there is a shared goal to improve healthcare and outcomes that are important to patients, and an increased awareness that the best way to deliver this is to work in partnership with patients. By working with patient communities, we can truly portray their diverse perspectives and experiences, and by collaborating across stakeholder groups (integrated with patients), we can capture the insights and information we need to transform healthcare.
